# Supplementary material for: A gradual transition toward categorical representations along the visual hierarchy during working memory, but not perception
Source: bioRxiv. 2024 Oct 7:2023.05.18.541327. Originally published 2023 May 18. Preprint. [Version 2] doi: 10.1101/2023.05.18.541327 (PMC10245673; doi:10.1101/2023.05.18.541327)
Supplement: Supplement 1 [file NIHPP2023.05.18.541327v2-supplement-1.pdf]

bioRxiv preprint doi: https://doi.org/10.1101/2023.05.18.541327; this version posted October 7, 2024. The copyright holder for this preprint (which was not certified by peer review) is the author/funder, who has granted bioRxiv a license to display the preprint in perpetuity. It is made available under aCC-BY 4.0 International license.

Supplementary Materials

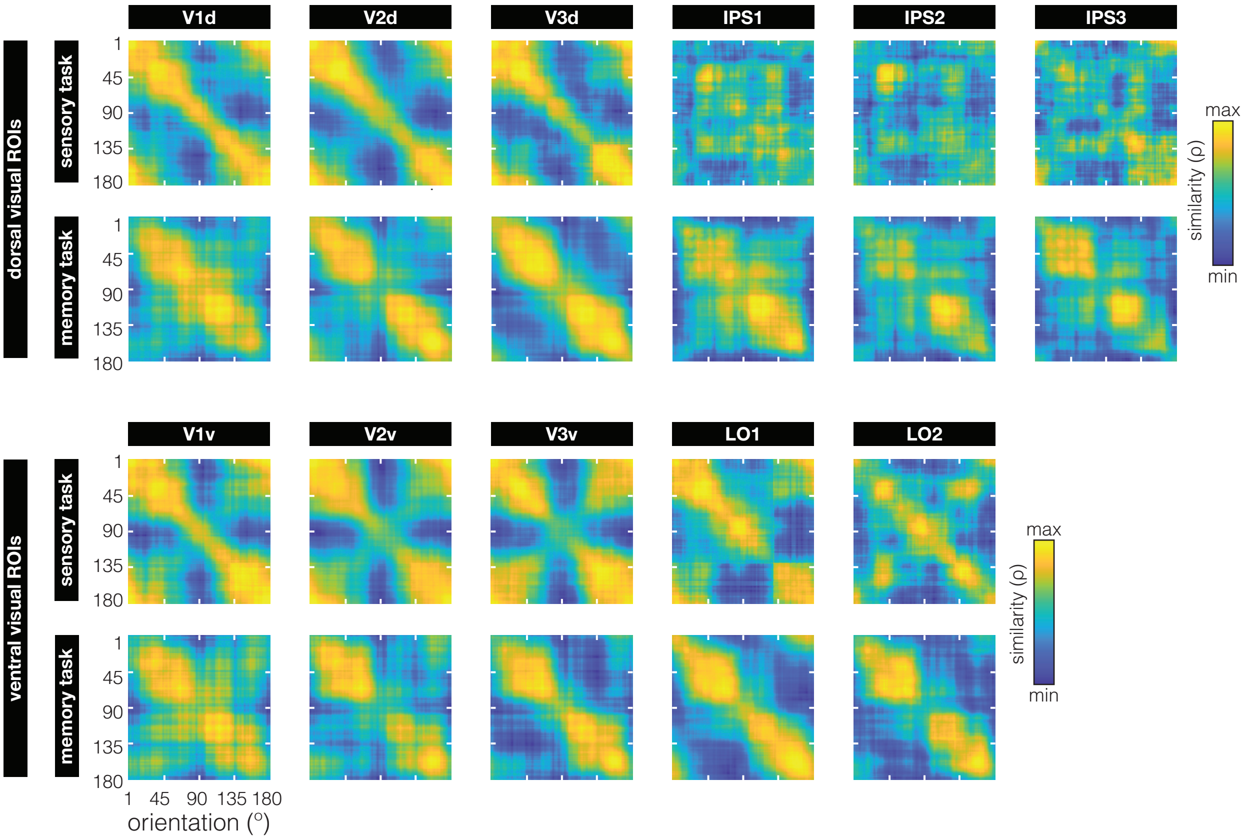

Supplementary Figure 1: Orientation representational geometry (as indexed by RSM's) during sensory perception and working memory for all retinotopically defined ROI's (across all participants) that were not already shown in Figure 1C. Here, ROI's are organized by whether they are located in the dorsal or ventral stream (top and bottom two rows, respectively). Early visual areas V1–V3 were split by their dorsal and ventral portions – used as input to the second-level RSA analysis (Figure 5 of the main text). Areas IPS1–3 (in the dorsal stream) and LO (in the ventral stream) were split based on their respective sub-portions – and similarly used as input to the second-level RSA analyses. All RSM's are scaled to the range of correlations within each subplot to ease visual comparison of representational structure.

bioRxiv preprint doi: https://doi.org/10.1101/2023.05.18.541327; this version posted October 7, 2024. The copyright holder for this preprint (which was not certified by peer review) is the author/funder, who has granted bioRxiv a license to display the preprint in perpetuity. It is made available under aCC-BY 4.0 International license.

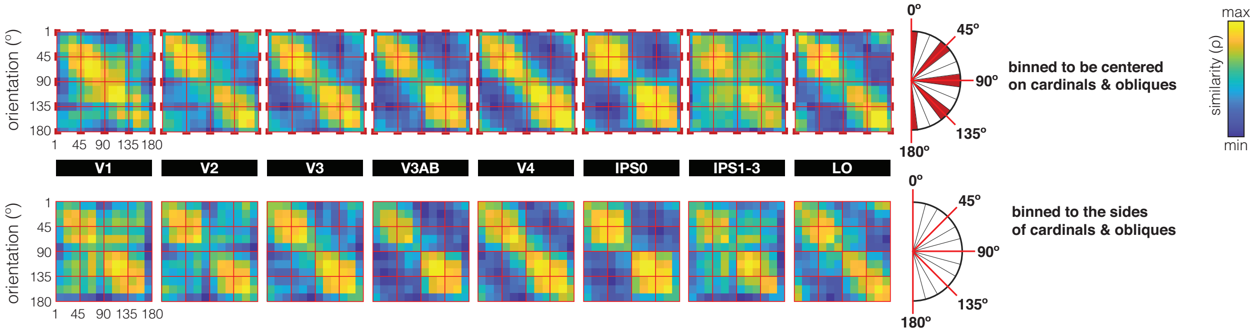

Supplementary Figure 2: With 180 possible target orientations, and a finite number of trials, some form of smoothing or binning is necessary for RSA to yield reliable correlations. In our main analysis we smooth over a window of ±10º, which could in theory impact the geometry around categorical boundaries (90º and 180º). In particular, it could induce some smearing of the categorical pattern observed in the memory task. To ensure that this pattern does not critically depend on the way trials are combined, here we show the data for the memory task binned (instead of smoothed) into 12 bins of 15º. On top, we show RSM's with bins *centered on* the 2 cardinals and the 2 obliques (see inset), meaning that the parts of orientation space highlighted in dark-red are bins that include a cardinal or an oblique orientation. On the bottom, we show the same analysis but with the bins shifted, such that they respect cardinal and oblique boundaries, and bins *fall on either side*. We observe that similarity in bins that include a cardinal (top row) is relatively low, presumably due to the relatively large psychological distance between orientations on different sides of a cardinal orientation, resulting in lower correlations. Nevertheless, there is relatively low similarity around cardinals *also* when we respect the categorical boundary (bottom row), implying these categorical effects are not impacted much by the specific binning approach. Overall, binning or smoothing do not drastically change the geometry (though of course, the resolution of the RSM is much lower with binning).

bioRxiv preprint doi: https://doi.org/10.1101/2023.05.18.541327; this version posted October 7, 2024. The copyright holder for this preprint (which was not certified by peer review) is the author/funder, who has granted bioRxiv a license to display the preprint in perpetuity. It is made available under aCC-BY 4.0 International license.

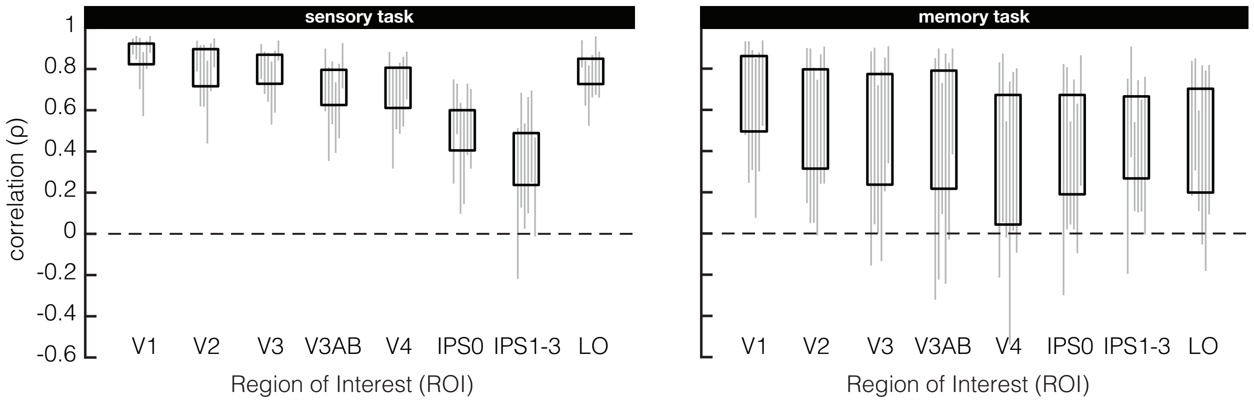

Supplementary Figure 3: Exact ranges of correlations in the RSM's from Figure 1C. To best show the representational structure for sensory and memory representations across ROI's, and to ease comparison between them, the RSM's in Figure 1C are scaled to the range (min-to-max) of correlations within each subplot. But the minimum and maximum correlations are not identical across subplots, therefore, correlation ranges across all participants (black rectangles) and individual participants (grey lines) are shown here for sensory (left) and memory (right) RSM's.

bioRxiv preprint doi: https://doi.org/10.1101/2023.05.18.541327; this version posted October 7, 2024. The copyright holder for this preprint (which was not certified by peer review) is the author/funder, who has granted bioRxiv a license to display the preprint in perpetuity. It is made available under aCC-BY 4.0 International license.

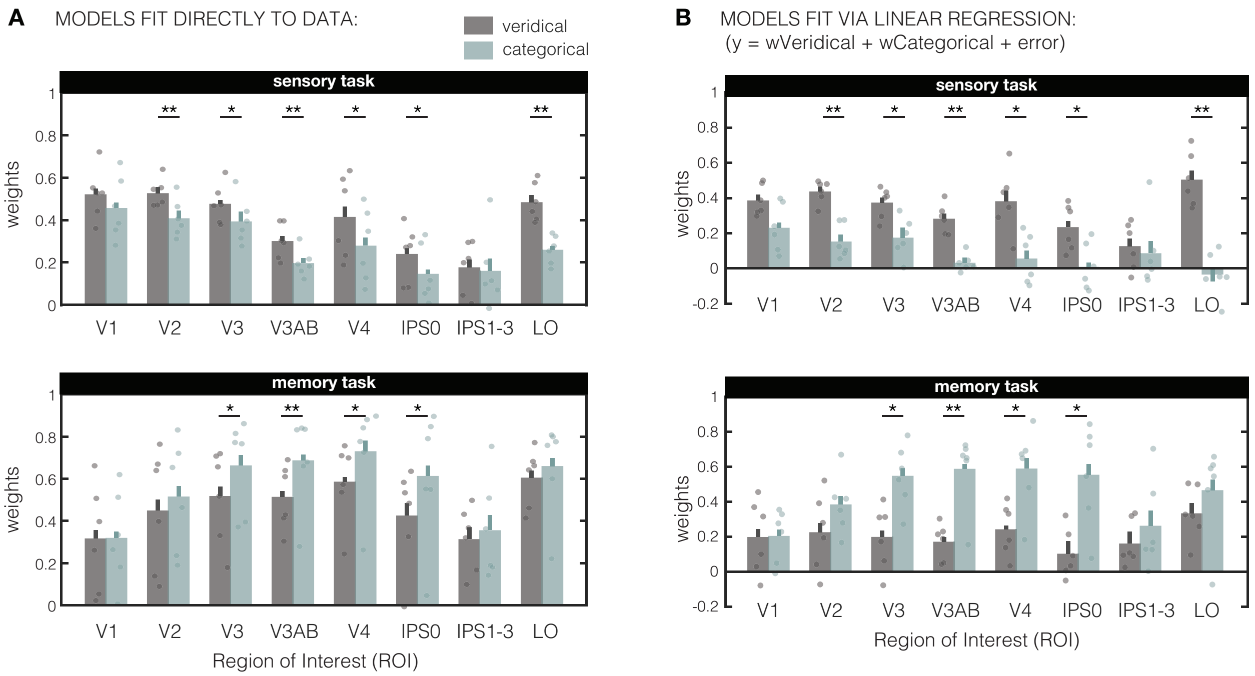

Supplementary Figure 4: Two alternative fitting approaches. (A) Model weights when fitting the veridical and categorical models directly to the RSM's (without first taking the residuals), and **(B)** model weights derived with a general linear regression (independent weights for each model). Irrespective of the fitting approach, the geometrical differences between our two tasks are captured by higher "veridical" weights in the sensory task, and more "categorical" weights in the memory task. For the "direct fitting" approach (in **A**) there is a significant 3-way interaction (model x ROI x task, $F_{(7,35)}$ = 2.413; p = 0.0398), which we followed up by post-hoc ANOVA's within the sensory and memory task separately. There is a main effect of model in both the sensory ($F_{(1,5)}$ = 40.26; p = 0.001) and memory ($F_{(1,5)}$ = 12.47; p = 0.017) tasks that is not the same in all ROI's (as indexed by model x ROI interactions for sensory $F_{(7,35)}$ = 3.262, p = 0.009 and memory $F_{(7,35)}$ = 2.791, p = 0.024 tasks). Similarly, for the general linear regression approach (in **B**) there is also a significant 3-way interaction (model x ROI x task, $F_{(7,35)}$ = 2.414; p = 0.0398), and main effects of model in both the sensory ($F_{(1,5)}$ = 40.28, p = 0.001) and memory ($F_{(1,5)}$ = 12.48, p = 0.017) tasks, and this difference between the models is not the same in all ROI's (as indexed by model x ROI interactions for both sensory $F_{(7,35)}$ = 3.26, p = 0.009, and memory $F_{(7,35)}$ = 2.79, p = 0.024 tasks). Asterisks indicate the significance level of post-hoc two-sided paired-sample t-tests (*p $\leq$ 0.05; **p $\leq$ 0.01) comparing the two models in each ROI.

bioRxiv preprint doi: https://doi.org/10.1101/2023.05.18.541327; this version posted October 7, 2024. The copyright holder for this preprint (which was not certified by peer review) is the author/funder, who has granted bioRxiv a license to display the preprint in perpetuity. It is made available under aCC-BY 4.0 International license.

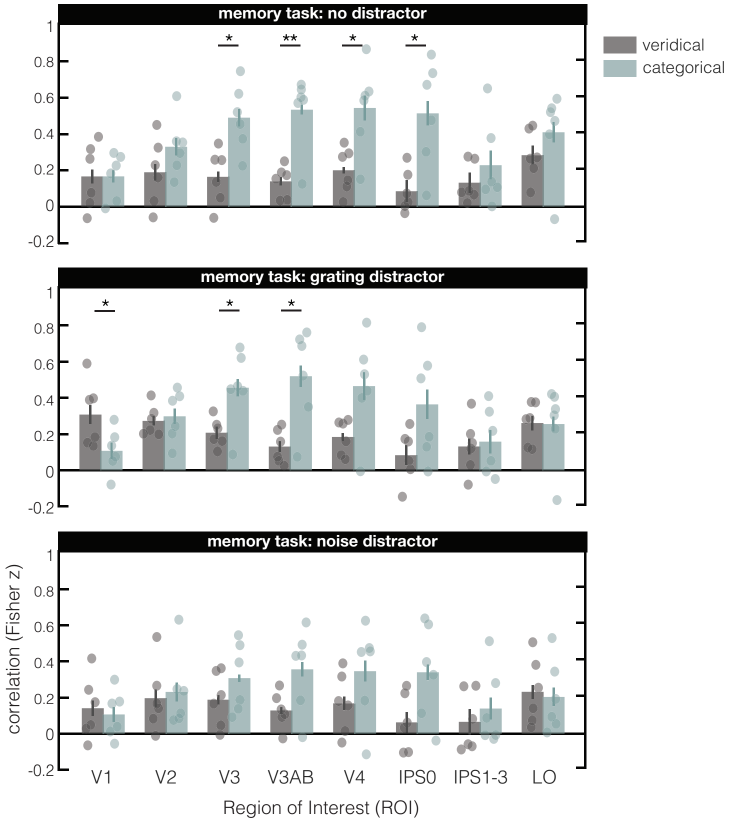

Supplementary Figure 5: Model fits for the 3 different working memory distractor conditions. Overall, the results split by condition are qualitatively similar to the main results across all trails (Figure 2D, bottom panel). Two-way ANOVA's comparing model and ROI showed that the categorical model did a better job at explaining the data in some of the ROI on trials without a distractor (model x ROI interaction: $F_{(7,35)}$ = 2.914; p = 0.016; main effect model: $F_{(1,5)}$ = 13.07; p = 0.015) and with a grating distractor (model x ROI interaction: $F_{(7,35)}$ = 4.3; p = 0.0016; main effect model: $F_{(1,5)}$ = 6.344; p = 0.053), indicating increasing differences between the veridical and categorical models along the visual hierarchy. While we see similar trends for the 108 trials with a noise distractor, these effects did not reach significance (model x ROI interaction: $F_{(7,35)}$ = 1.812; p = 0.116; main effect model: $F_{(1,5)}$ = 1.335; p = 0.3). Nevertheless, despite using only 1/3rd of the data in each of these sub-plots, the pattern in the data is highly consistent. Asterisks indicate the significance level of post-hoc two-sided paired-sample t-tests (*p $\leq$ 0.05; **p $\leq$ 0.01) comparing the two models in each ROI.

bioRxiv preprint doi: https://doi.org/10.1101/2023.05.18.541327; this version posted October 7, 2024. The copyright holder for this preprint (which was not certified by peer review) is the author/funder, who has granted bioRxiv a license to display the preprint in perpetuity. It is made available under aCC-BY 4.0 International license.

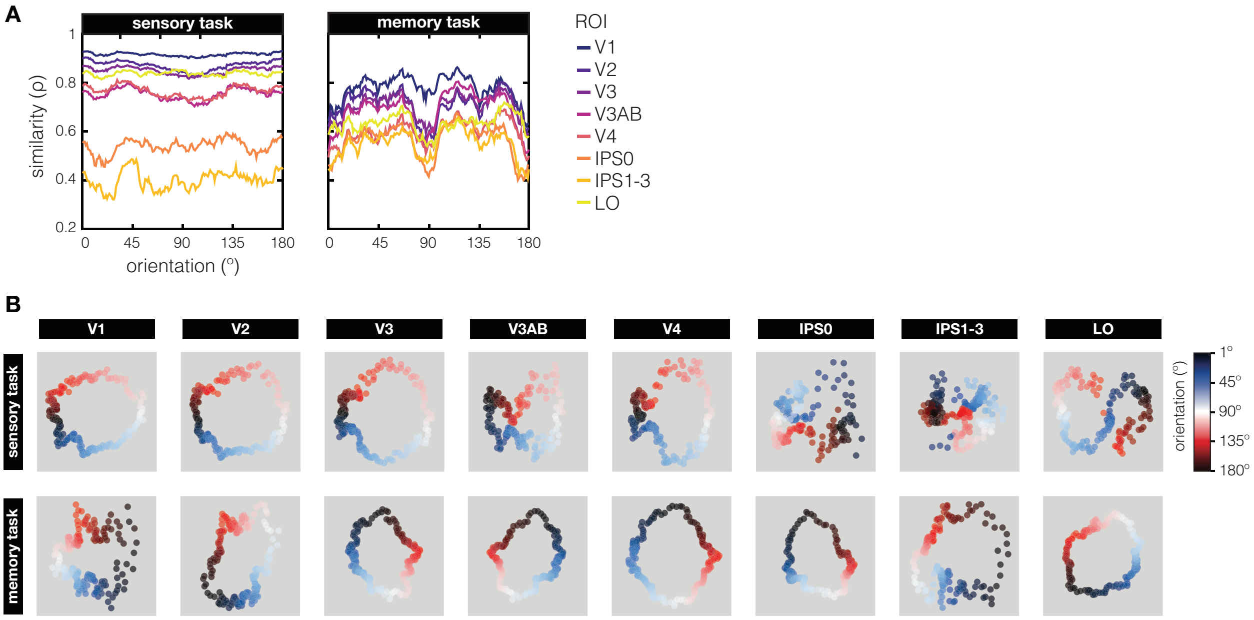

Supplementary Figure 6: Orientation inhomogeneities of the representational geometry (A) To examine the inhomogeneity or representational similarity throughout orientation space, we plot the diagonals of the RSM's from in Figure 1C. During the sensory task, we see that similarity tends to be relatively high around vertical orientations (0º/180º) compared to horizontal orientations (90º). For both tasks, oblique orientations are represented relatively more similar, and cardinals less similar. This "oblique" like effect is much exacerbated in the memory task compared to the sensory task. **(B)** We use multidimensional scaling (MDS) to projects high dimensional response patterns into 2 dimensions, in order to better visualize of how orientation space is represented. During the sensory task there is an orderly geometrical progression of orientation space, with the highest similarity between adjacent orientations (and some clustering around cardinal orientations, especially 180º) in early visual areas V1–V3. There's also a "pinching" of orientation space around the obliques (45º and 135º become very similar) in more anterior visual areas V3AB–IPS and LO. During the memory task, the orientation space geometry remains circular in all ROI's, with notable clustering of similarity around the obliques.

bioRxiv preprint doi: https://doi.org/10.1101/2023.05.18.541327; this version posted October 7, 2024. The copyright holder for this preprint (which was not certified by peer review) is the author/funder, who has granted bioRxiv a license to display the preprint in perpetuity. It is made available under aCC-BY 4.0 International license.

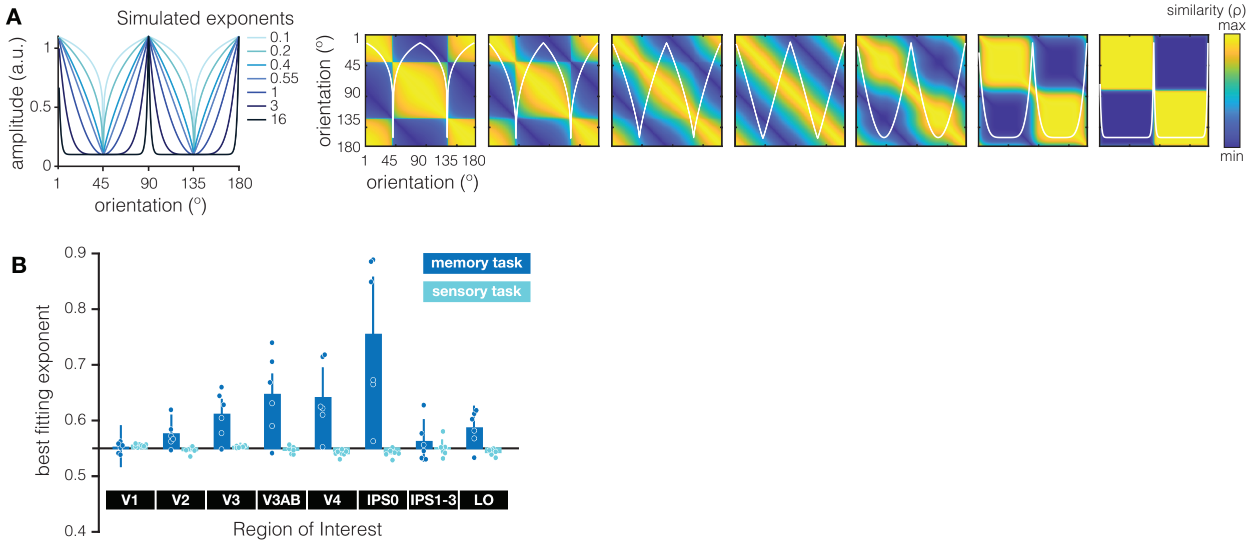

Supplementary Figure 7: Alternative model based on psychological distance. (A) An alternative way to model the sensory and memory task RSM's is to vary the degree of similarity that can be expected at cardinals or at obliques. By changing the exponent in the input statistics function $f(x) = \left||\sin x| - 1\right|^{exponent} + 0.1$ to a free parameter, and using the psychological distance between every pair of orientations (as in the categorical model), we can create a family of input statistics functions (left panel) that modulate the shape of the model RSM's (right panels) such that we can span any orientation anisotropy ranging from highest similarity around cardinals to highest similarity around obliques (similar to the modeling approach in [57]). Each of the input functions in the left panel matches an RSM in the right panels (with the input function overlaid in white). At an exponent of 0.55 we approximate a uniform diagonal RSM, or a "physical" model of orientation space. Note that by modulating the shape of the input function in this manner, we can retrieve models that look very similar to our veridical model (e.g., exponent = 0.4), and a model identical to our categorical model (exponent = 2) in this parametric RSM space. **(B)** We plot the best fitting exponent for the input function for all ROI's (x-axis) and separately for the memory (dark blue) and sensory (light blue) tasks, and show that those differ significantly (ROI x task interaction: $F_{(7,35)} = 9.658$; $p < 0.001$). For the sensory task the best fitting exponent stays close to 0.55 for all ROI's, indicating that an RSM with a close-to uniform diagonal fits the data well. That said, the exponent does differ across ROI's (main effect of roi, $F_{(7,35)} = 3.307$; $p = 0.008$), showing that a model with slightly higher similarity around obliques (exponent > 0.55) does better in for example V1, while a model with slightly higher similarity at cardinals (exponent between 0 and 0.55) does better at for example V4. For the memory task we see a gradual increase in the exponent along the visual hierarchy (up to and including IPS0), indicating that a model with increasingly stronger similarity around oblique orientations (i.e., increasingly stronger categorization) is better at explaining the memory geometry for more anterior ROI's (main effect of roi, $F_{(7,35)} = 9.213$; $p < 0.001$). Best fitting exponents for individual subjects are shown as dots, and error bars indicate $\pm$ 1 within-subject SEM.

bioRxiv preprint doi: https://doi.org/10.1101/2023.05.18.541327; this version posted October 7, 2024. The copyright holder for this preprint (which was not certified by peer review) is the author/funder, who has granted bioRxiv a license to display the preprint in perpetuity. It is made available under aCC-BY 4.0 International license.

		V1	V2	V3	V3AB	V4	IPS0	IPS1–3	LO
theoretical	sensory	t=2.426 p=0.059	t=5.274 p=0.003	t=3.099 p=0.027	t=5.792 p=0.002	t=3.247 p=0.023	t=2.584 p=0.049	t=0.26 p=0.805	t=4.42 p=0.006
	memory	t<0.001 p=0.999	t=1.79 p=0.133	t=3.424 p=0.019	t=4.398 p=0.007	t=2.812 p=0.037	t=3.233 p=0.023	t=0.824 p=0.448	t=1.1 p=0.321
behavior	sensory	t=8.204 p<0.001	t=6.772 p=0.001	t=5.178 p=0.004	t=5.383 p=0.003	t=5.062 p=0.004	t=3.221 p=0.023	t=1.287 p=0.255	t=6.28 p=0.002
	memory	t=0.067 p=0.949	t=1.534 p=0.186	t=3.131 p=0.026	t=3.558 p=0.016	t=4.257 p=0.008	t=2.529 p=0.053	t=0.463 p=0.663	t=1.096 p=0.323

Supplementary Table 1: Post-hoc statistics for two-sided paired t-tests from the theoretical input function based on the statistics in the natural world (in green) and from the psychophysical input function based on independent behavioral measurements (in blue). All significant cells are colored in a lighter shade for the purpose of quick visualization.
