# Supplementary material for: A gradual transition from veridical to categorical representations along the visual hierarchy during working memory, but not perception
Source: bioRxiv. 2023 May 18:2023.05.18.541327. Preprint. [Version 1] doi: 10.1101/2023.05.18.541327 (PMC10245673; doi:10.1101/2023.05.18.541327)
Supplement: Supplementary file 1 [file NIHPP2023.05.18.541327v1-supplement-1.pdf]

bioRxiv preprint doi: https://doi.org/10.1101/2023.05.18.541327; this version posted May 18, 2023. The copyright holder for this preprint (which was not certified by peer review) is the author/funder, who has granted bioRxiv a license to display the preprint in perpetuity. It is made available under aCC-BY 4.0 International license.

Supplementary Materials

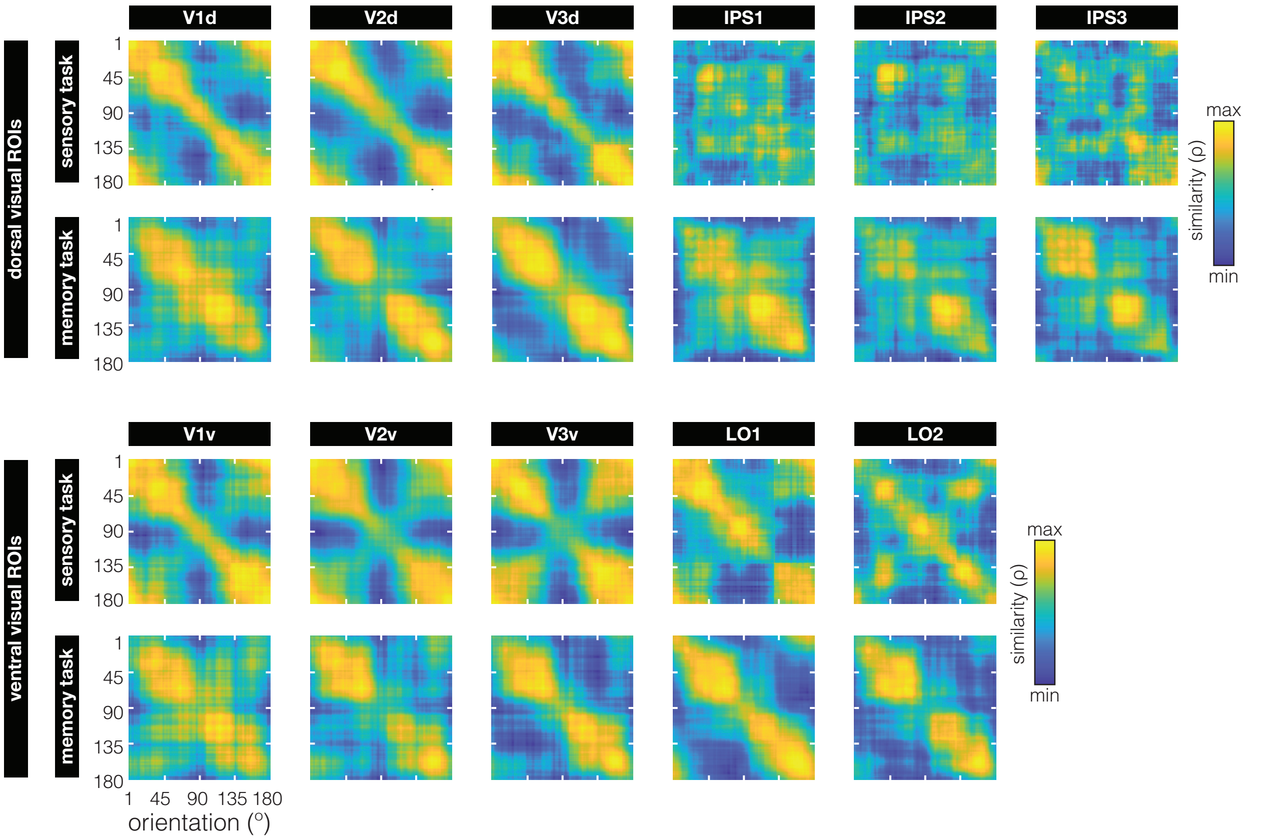

Supplementary Figure 1: Orientation representation (as indexed by RSM's) during sensory perception and working memory for all retinotopically defined ROI's (across all participants) that were not already shown in Figure 1C. ROI's are organized by whether they are located in the dorsal or ventral stream (top and bottom two rows, respectively). Early visual areas V1–V3 were split by their dorsal and ventral portions – used as input to the second-level RSA analyses (Figure 3). Areas IPS1–3 (in the dorsal stream) and LO (in the ventral stream) were split based on their respective sub-portions – and similarly used as input to the second-level RSA analyses. All RSM's are scaled to the range of correlations within each subplot to ease visual comparison of representational structure.

bioRxiv preprint doi: https://doi.org/10.1101/2023.05.18.541327; this version posted May 18, 2023. The copyright holder for this preprint (which was not certified by peer review) is the author/funder, who has granted bioRxiv a license to display the preprint in perpetuity. It is made available under aCC-BY 4.0 International license.

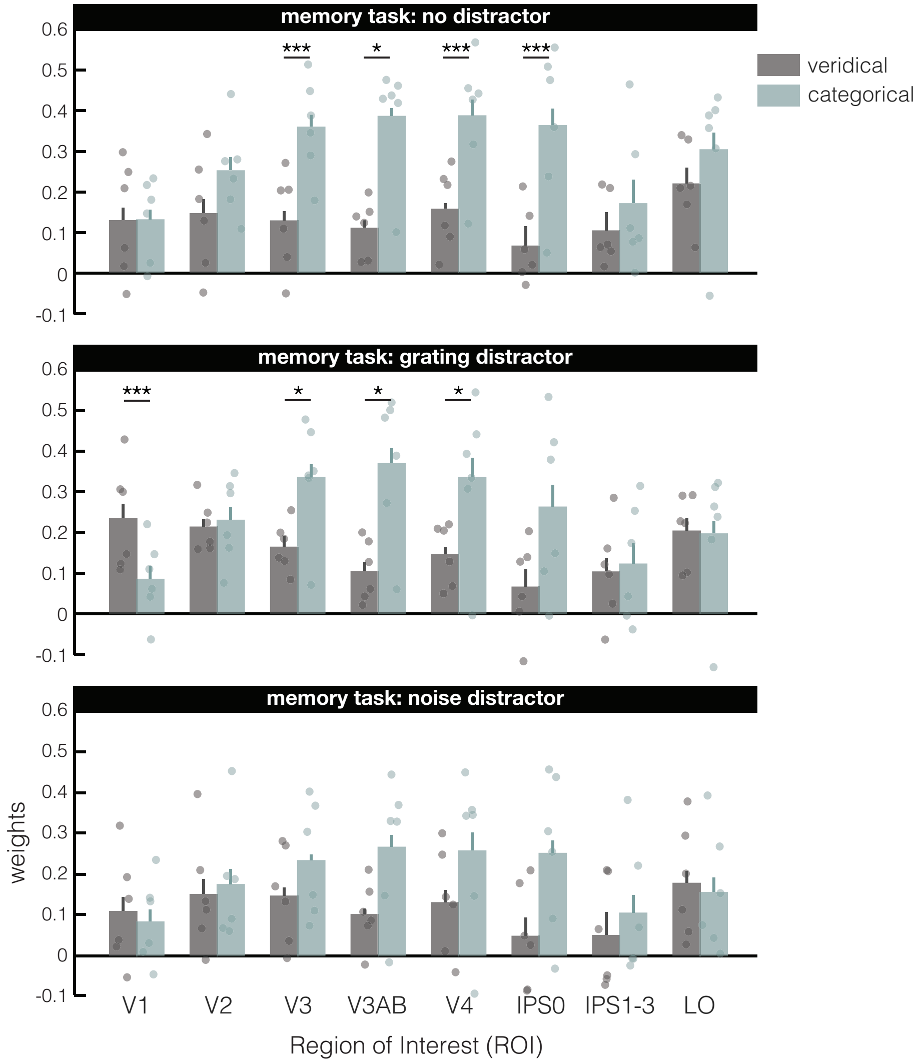

Supplementary Figure 2: Model fits for the 3 different working memory distractor conditions. Overall, the results split by condition are qualitatively similar to the main results across all trails (Figure 2D, bottom panel). The two-way ANOVA's comparing model and ROI showed that the categorical model did a better job at explaining the data without a distractor (main effect model: $F_{(1,5)} = 12.29$; $p = 0.017$), and marginally so with a grating distractor ($F_{(1,5)} = 5.55$; $p = 0.065$) presented during the delay. Both n-distractor and grating-distractor conditions also had significant interactions ($F_{(7,35)} = 2.778$; $p = 0.017$ and $F_{(7,35)} = 4.234$; $p = 0.002$), indicating increasing differences between the veridical and categorical models along the visual hierarchy. For the 108 trials during which a noise distractor was presented during the delay, neither the main effect of model ($F_{(1,5)} = 1.227$; $p = 0.318$), nor the interaction between model and ROI ($F_{(7,35)} = 1.745$; $p = 0.13$) reached statistical significance. Nevertheless, despite using only 1/3rd of the data in each of these sub-plots, the pattern in the data do not change

bioRxiv preprint doi: https://doi.org/10.1101/2023.05.18.541327; this version posted May 18, 2023. The copyright holder for this preprint (which was not certified by peer review) is the author/funder, who has granted bioRxiv a license to display the preprint in perpetuity. It is made available under aCC-BY 4.0 International license.

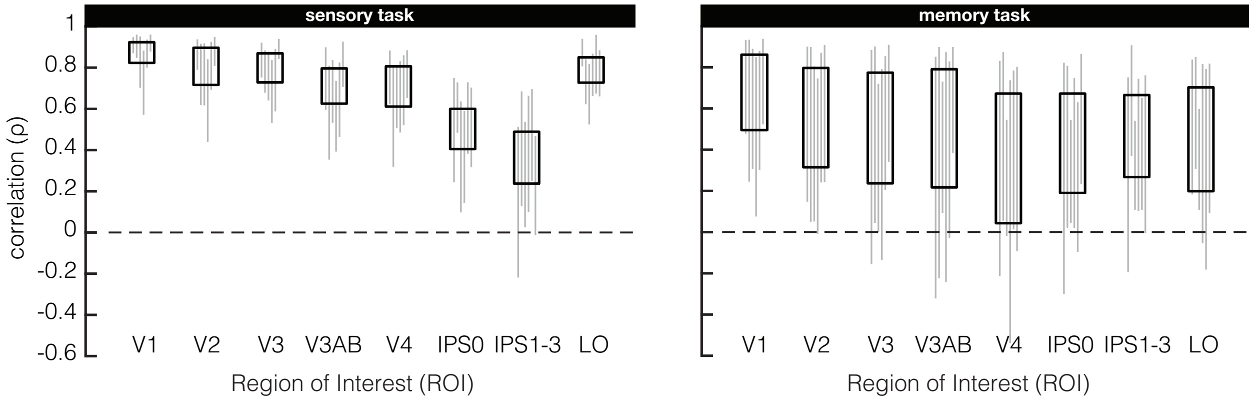

Supplementary Figure 3: Exact ranges of correlations in the RSM's from Figure 1C. To best show the representational structure for sensory and memory representations across ROI's, and to ease comparison between them, the RSM's in Figure 1C are scaled to the range (min-to-max) of correlations within each subplot. But the minimum and maximum correlations are not identical across subplots, therefore, correlation ranges across all participants (black rectangles) and individual participants (grey lines) are shown here for sensory (left) and memory (right) RSM's.

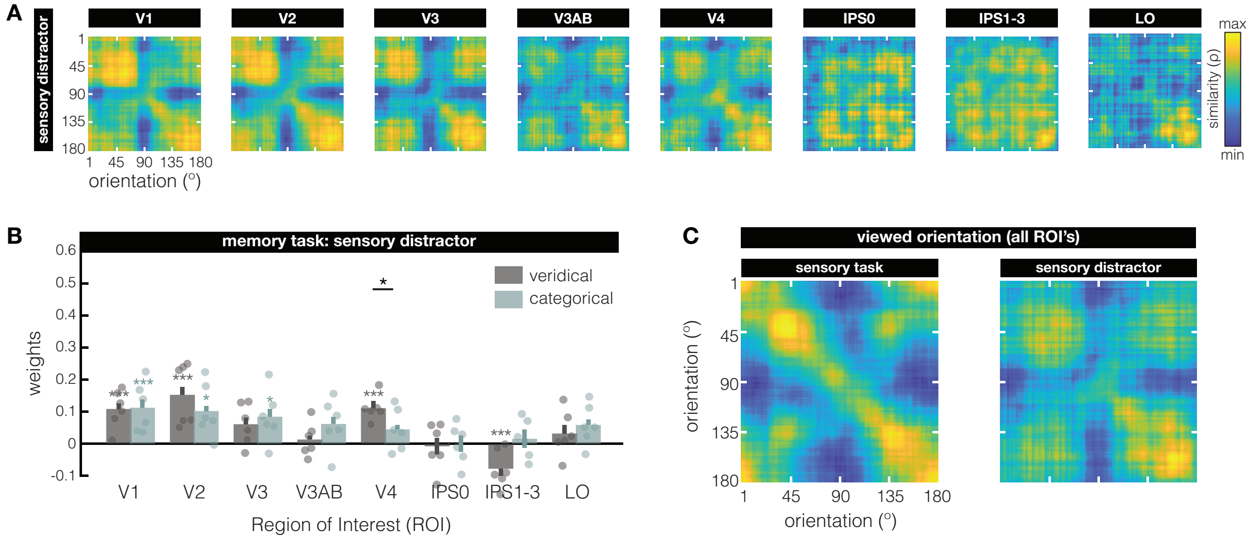

Supplementary Figure 4: Orientation representations of viewed gratings shown during the working memory delay (A) To test the generalizability of our finding that sensory inputs are represented in a predominantly veridical manner (Figure 2D, top), we constructed RSM's from voxel responses to directly viewed gratings presented as distractors during the delay of our memory task (for retinotopically defined ROI's and across all participants). The grating distractor was shown for 11s during the working memory delay on one-third of trials. Importantly, orientations of the remembered and distractor gratings were random and independent, allowing us to also examine the representational similarity structure of the distractor. In early visual areas, the RSM's show a diagonal pattern (with highest similarity around 180°) combined with some degree of clustering around obliques. Indeed, the modeling results in **(B)** show that both the veridical and categorical models explain the representational pattern to some extent. There is a significant interaction between ROI and model ($F_{(7,35)}$ = 5.21; p = 0.025), indicating that the two models capture the data differently in different ROI's. Only in area V4 did the veridical model outperform the categorical model, but there was no difference between the two models overall (main effect of model, $F_{(1,5)}$ = 1.054; p = 0.308) in V1, V2, and V4 (and an opposite effect in IPS1-3). **(C)** Here we show RSM's of viewed orientations under two different contexts side-by-side (across all visual ROI's). During the sensory task, participants had to attend the grating and detect contrast changes (left). When the sensory distractor was on the screen, participants had to ignore the grating while they were performing a concurrent VWM task (right). In addition to differences in attentional state, the number of trials collected in these different settings also differed (300+ for the sensory task, 108 for the sensory distractor shown during the memory task), likely causing differences in the signal-to-noise ratios. Thus, the differences between these two perceptual contexts may be due to multiple reasons.

bioRxiv preprint doi: https://doi.org/10.1101/2023.05.18.541327; this version posted May 18, 2023. The copyright holder for this preprint (which was not certified by peer review) is the author/funder, who has granted bioRxiv a license to display the preprint in perpetuity. It is made available under aCC-BY 4.0 International license.

		V1	V2	V3	V3AB	V4	IPS0	IPS1–3	LO
theoretical	sensory	t=2.4434 p=0.0319	t=5.1586 p<0.001	t=3.0225 p=0.0312	t=5.9727 p<0.001	t=3.3920 p=0.0307	t=2.6689 p=0.0305	t=0.3260 p=0.7496	t=4.7392 p<0.001
	memory	t=0.0319 p=0.9377	t=2.0768 p=0.0620	t=3.5862 p<0.001	t=4.0215 p=0.0317	t=2.9295 p<0.001	t=3.6337 p<0.001	t=0.7569 p=0.4694	t=0.8297 p=0.4059
behavior	sensory	t=4.3757 p<0.001	t=5.0921 p<0.001	t=4.1982 p<0.001	t=2.4459 p=0.0303	t=2.8264 p=0.0309	t=0.7673 p=0.4686	t=0.0201 p=0.969	t=2.6235 p=0.0318
	memory	t=3.2622 p=0.0315	t=4.1277 p<0.001	t=5.8139 p<0.001	t=6.0233 p<0.001	t=7.3358 p<0.001	t=5.3503 p<0.001	t=2.4268 p<0.001	t=1.6610 p=0.1568

Supplementary Table 1: Post-hoc statistics for two-sided paired t-tests from the theoretical input function based on the statistics in the natural world (in green) and from the psychophysical input function based on independent behavioral measurements (in blue). All significant cells are colored in a lighter shade for the purpose of quick visualization.
